# Application of Polymer Hydrogels in the Prevention of Postoperative Adhesion: A Review

**DOI:** 10.3390/gels9020098

**Published:** 2023-01-23

**Authors:** Jie Cai, Jiaming Guo, Shige Wang

**Affiliations:** 1 School of Materials and Chemistry, University of Shanghai for Science and Technology, No. 516 Jungong Road, Shanghai 200093, China; 2 Department of Radiation Medicine, College of Naval Medicine, Naval Medical University, No. 800 Xiangyin Road, Shanghai 200433, China

**Keywords:** hydrogel, postoperative adhesion, biocompatibility, biodegradability, drug delivery

## Abstract

Postoperative adhesion is a common post-surgery complication formed between the surface of the body cavity, ranging from a layer of connective tissue to a fibrous bridge containing blood vessels and nerve tissue. Despite achieving a lot of progress, the mechanisms of adhesion formation still need to be further studied. In addition, few current treatments are consistently effective in the prevention of postoperative adhesion. Hydrogel is a kind of water-expanding crosslinked hydrophilic polymer network generated by a simple reaction of one or more monomers. Due to the porous structure, hydrogels can load different drugs and control the drug release kinetics. Evidence from existing studies has confirmed the feasibility and superiority of using hydrogels to counter postoperative adhesions, primarily due to their outstanding antifouling ability. In this review, the current research status of hydrogels as anti-adhesion barriers is summarized, the character of hydrogels in the prevention of postoperative adhesion is briefly introduced, and future research directions are discussed.

## 1. Introduction

**Figure 1:**
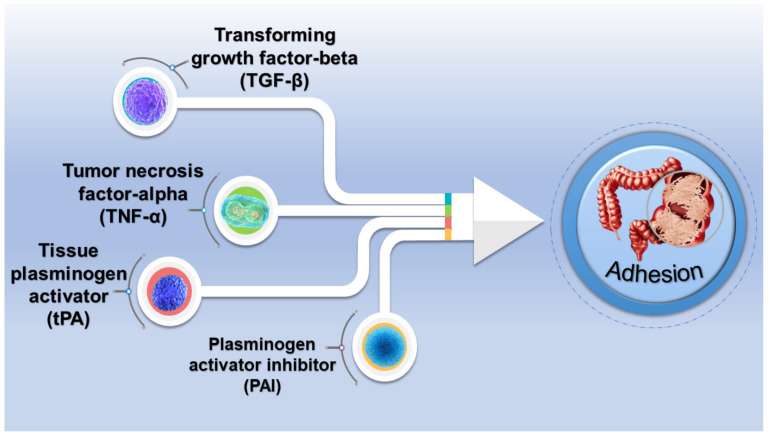
Some soluble mediators in adhesion formation.

Postoperative adhesion is a prevalent complication after surgery, which often leads to an increase in the morbidity and mortality of patients. It is reported that pathological adhesion occurs in 95% of surgeries, regardless of the surgical operation or the surgical location in the body [1]. Postoperative adhesions are pathological adhesions formed between the surface of the body cavity, ranging from a layer of connective tissue to a fibrous bridge containing nerve tissue and blood vessels [2,3]. The majority of severe chronic pain, organ dysfunction, etc. are related to the development of fibrous adhesions. In order to prevent postoperative adhesions, patients need to perform additional operations, which increases their medical costs and physical burden. The formation of postoperative adhesions mainly involves the fibrinolytic inhibition and degradation of the extracellular matrix, the evolution of inflammatory responses, and the formation of tissue hypoxia [4]. Macrophages, eosinophils, erythrocytes, mast cells, and fibroblasts are the primary components of adhesions. The cell population of the adhesions will change to polymorphonuclear leukocytes and fibroblasts as the adhesions gradually mature [5,6,7]. There are also factors critical to the regulation of inflammatory and immune responses, tissue remodeling, and angiogenesis (Figure 1). Therefore, understanding the influence of cells and factors on adhesion formation is important to developing successful prevention strategies.

Even though great efforts have been engaged in the mechanism study, postoperative adhesions remain a perplexing surgical challenge today. With the rapid development of molecular biology, oncology, pharmacology, and materials science, combination therapy has attracted extensive attention [8]. Further research and progressive understanding of nanoparticles, natural polymers, synthetic polymers, and hydrogels by researchers in recent years have opened up new directions in the field of postoperative adhesions [9,10,11]. At present, surgical relief, drug inhibition, biological materials, and physical barriers have been recruited to treat postoperative adhesions [12,13,14]. Hydrogels with a perfect performance show enormous potential in biomedical applications, such as wound dressings [15,16], hemostatic patches [17,18,19,20], drug delivery [21,22,23], anti-adhesion barriers [24,25,26], therapeutics [27,28,29], etc. Injectable hydrogel is a promising material because of its ability to target anchor defects deep in the tissue and its low invasiveness and safety. This hydrogel can be rapidly transformed from a precursor to a hydrogel under some environmental factors, such as temperature change, pH, or ionic strength change. Therefore, hydrogels are also frequently used in tissue engineering and wound dressings due to their water retention and the native extracellular matrix (ECM) homogeneity, and wound-healing hydrogels can not only protect the affected area but can also promote healing of the wound area.

Evidence from existing studies has confirmed the feasibility and superiority of using hydrogels to prevent postoperative adhesions [30,31]. This is because hydrogels have admirable biocompatibility and biodegradability [32]. In addition, due to their porous structure, hydrogels can load different drugs and control the drug release kinetics. Moreover, hydrogels have an excellent antifouling ability, which can prevent the adhesion of proteins and cells on the surface [33,34]. In this review, we first give a brief introduction to postoperative adhesions and an overview of hydrogels, including their definition, classification, synthesis method, and application. Further, the advantages of hydrogels for postoperative adhesion, including biocompatibility and biodegradability, delivery performance, and adhesion performance, are then summarized. Finally, the critical roles of hydrogels in postoperative adhesion prevention are highlighted, and future research directions are discussed.

## 2. Overview of Hydrogel

### 2.1. Definition of Hydrogel

Hydrogel, also known as a category of soft substance, is a network of hydrophilic polymers generated by the reaction of different monomers [35,36]. Biomacromolecular hydrogels are formed by a network of polymer chains interacting with water or biological fluids through capillary, osmotic, and hydrodynamic interactions, which balance each other and lead to the expansion of the chain network. This equilibrium state depends on the magnitude of these opposite effects, which determines the internal transport, diffusion characteristics, and mechanical strength [37,38]. Over the past few decades, hydrogels have received considerable attention because of their extraordinary promise in diverse fields. Due to their superb water retention capabilities, hydrogels have a flexibility that mimics the native ECM, which can support cell proliferation and migration and control the release of growth factors or drugs. Moreover, as soft materials, hydrogels have the appropriate roughness and lead to a minimal mechanical stimulation of the surrounding tissue, which further supports the cell proliferation and diffusion of nutrition [14,39]. Accordingly, these above features give hydrogels an incomparable technical potential in the biomedical area.

### 2.2. Classification of Hydrogels

(1) Natural hydrogels and synthetic hydrogels

The water absorption capacity of hydrogels generally comes from the hydrophilic functional groups connected to the main chain of polymers. In contrast, their anti-solubility comes principally from the network chains’ crosslinking. The organization, composition, degradability, chemical structure, biofunctionality, and physical properties of polymeric hydrogels can be readily engineered [35,40]. Based on the source of the polymer, hydrogels can be divided into natural and synthetic hydrogels [10]. Natural hydrogels can be composed of cellulose, starch, hyaluronic acid (HA), alginic acid, chitosan, collagen, poly (L-glutamic acid), poly (L-lysine), etc. Synthetic hydrogels can be made up of acrylic acid, polyacrylic acid, and polyacrylamide. Natural polymer hydrogels are mostly biocompatible and minimally stimulate inflammatory or immune responses to host tissues. Therefore, they have been extensively studied as biomaterials for tissue engineering and biomedicine [41,42]. Unfortunately, the strength and toughness of most natural polymers are poor and occasionally cannot meet clinical requirements. By contrast, synthetic polymers have significant advantages. For example, they have well-defined structures that can be modified to optimize and acquire the desired functionality [43,44]. Some of the classifications of hydrogels and their simple advantages and disadvantages are detailed in Table 1.

(2) Physical and chemical crosslinking hydrogels

Further, hydrogels can be divided into physical and chemical crosslinking hydrogels as per the crosslinking degrees [45,46,47]. One of the advantages and significance of physical crosslinking hydrogels is that they are easy to produce, since a crosslinking agent is not necessary. The primary driving force of physical hydrogels could be hydrogen bonds, van der Waals forces, or electrostatic forces. The design of physical crosslinking hydrogels is susceptible to different factors, such as polymer concentrations, pH of the polymer solutions, and temperature of surroundings, which lead to the formation of gels with various gel times, internal network apertures, chemical functionalization, and degradation times. Considering that they can be transformed into solutions upon heating, physical hydrogels are also called pseudo-gels or thermally reversible gels [48]. Physical crosslinking hydrogels can be synthesized by freezing and thawing gelling [42,49], stereoscopic complexing [50,51], ion interaction gelling [52,53], and hydrogenbonding gelling [54,55,56]. Up to now, physical crosslinking hydrogels have attracted substantial attention in pharmaceutical, food, and biomedical applications.

The typical driving force of chemical crosslinking hydrogels is chemical bonds [57,58]. Chemical crosslinking hydrogels are stable and hard to dissolve even under a high temperature. Nevertheless, they can be dissolved when using their solvents as their counterparts to break the bond [59,60,61]. Moreover, these chemical bonds endow the chemical crosslinking hydrogels with durable mechanical strength [60,62,63]. The primary methods of obtaining chemical crosslinking hydrogels include: (1) using a chemical crosslinking agent [64,65], (2) high-energy radiation [66], (3) free radical polymerization [67,68], (4) condensation reaction polymerization [69,70], (5) an enzymatic reaction [71,72,73,74], etc. The chemical crosslinking hydrogel is a permanent three-dimensional network polymer and usually shows a retarded degradation behavior.

(3) Homopolymer hydrogels, copolymerized hydrogels, and polymer hydrogels

According to the polymer composition, hydrogels can be divided into homopolymer hydrogels, copolymerized hydrogels, and polymer hydrogels. Homopolymer hydrogels, which mainly refer to the polymer network derived from a single monomer, can also have crosslinked skeleton structures due to different monomer properties and polymerization techniques [75,76]. Copolymerized hydrogels, which are composed of different monomer species with at least one hydrophilic component, are arranged along the chain of the polymer network in random, block, or alternate configurations [76,77,78,79,80]. Polymer hydrogels, which exist in the form of a crosslinked network, consist of independent polymers.

(4) Solid hydrogels, semi-solid hydrogels, and liquid hydrogels

Additionally, based on their physical state, hydrogels can be classified into solid, semi-solid, and liquid hydrogels. Solid hydrogels are typically robust and flexible and can simulate the physicochemical, electrical, and biological properties of biological tissues by simulating complex tissue structures and providing the necessary cellular microenvironment [81]. Solid hydrogels have a strong crosslinked network at room temperature and can reveal swelling properties in water, buffer solutions, or biological fluids. Because of these unique properties, they can be used to prepare hydrogels for biomedical applications [82,83]. Semi-solid hydrogels are typically synthesized by interfacial forces such as van der Waals forces, electrostatic forces, and hydrogen bonds [84,85,86]. They usually have soft tissue networks and strong adhesion, giving them appealing promise for extended administration and effective dose application in biomedical fields via ocular, oral, rectal, nasal, vaginal, and sublingual approaches. Moreover, semi-solid hydrogels also have good wetting, adsorption, and desorption properties. Liquid hydrogels usually behave as a liquid intermediate phase at room temperature with an elastic phase similar to that of soft tissues [87,88,89]. Liquid hydrogels have biocompatibility and functionality, and they can adapt to the shape of their surroundings. Most liquid hydrogels possess an excellent loading capacity, which is qualified in the loading of organics, inorganics, drugs, proteins, and cells. Moreover, the liquid hydrogel is injectable; therefore, it can be readily injected into the target domains.

**Table 1:** Classification and properties of hydrogels.

Basis	Classify	Advantages	Disadvantages	References
Polymer sources	Natural hydrogel	Biocompatible, low irritation	Low strength, toughness	[41]
	Synthetic hydrogel	High gel strength, long life performance	Low biocompatibility, irritation to tissues	[43]
Crosslinking degree	Physical crosslinking hydrogels	Easy to produce, high biosafety, reversible	Susceptible to outside influences, lack of stability	[47]
	Chemical crosslinking hydrogels	Stable, long-lasting mechanical strength	Irreversible, low biocompatibility, difficult to degrade	[57]
Polymer composition	Homopolymer hydrogels	Pure product, low impurities	Limited performance improvements	[75]
	Copolymerized hydrogels	Excellent properties	High cost of raw materials	[78]
	Polymer hydrogels	High flexibility, diverse properties	Difficult to synthesize	[16,80]
Physical state	Solid hydrogel	Robust and flexible, with a strong crosslinked network structure	Weak tissue adhesion	[29,53,84,90]
	Semi-solid hydrogel	Strong adhesion, good wetting, adsorption properties	Synthetic complexity	[29,86]
	Liquid hydrogel	Biocompatible, functional, excellent loading capacity	Hard to surface modify	[89]

## 3. Advantages of Hydrogels for the Prevention of Postoperative Adhesion

### 3.1. Biocompatibility and Biodegradability

It is hoped that the hydrogels used for postoperative adhesions should have both splendid biocompatibility and certain biodegradability. Biocompatibility primarily implies histocompatibility and hemocompatibility. Histocompatibility refers to the mutual adaptability of materials and human tissues and organs. Hemocompatibility refers to the notion that the materials cause no adverse reactions, such as coagulation or hemolysis, to blood cells [91,92]. In general, biocompatibility is related to the structure and properties of the material, such as microphase structure, hydrophilicity, hydrophobicity, surface charge, etc. [93,94]. Typically, the hydrogels used as anti-adhesion barriers are made of natural or adaptive polymers, which have good biocompatibility to meet the requirements of postoperative anti-adhesion hydrogels. However, the degradation rate of certain kinds of natural polymers is fast, resulting in insufficient anti-adhesion. In contrast, the degradation velocity of synthetic polymers is slower than natural polymers and thus has a longer retention duration, which can facilitate the complete prevention of postoperative adhesions. Therefore, hydrogels are expected to have enhanced anti-adhesion performance. Moreover, hydrogel exudates, such as residual monomers, impurities, additives, degradants, and metabolites, may also trigger a series of tissue reactions. Therefore, it is important to test the biocompatibility of hydrogels when using them to prevent postoperative adhesions. In the following, some examples of biocompatible natural polymers are detailed.

#### 3.1.1. Hyaluronic Acid

HA is a linear biocompatible natural polymer composed of repeated units of N-acetylglucosamine and gluconic acid [95]. Because HA can crosslink with other molecules, the use of HA in the hydrogel is beneficial for obtaining desired mechanical properties. Moreover, since it is biodegradable, HA is often mixed with other materials to extend its stability. For example, Babuska et al. prepared polyvinyl alcohol (PVA) and HA composite scaffold by in situ synthesis and physical mixing using prefabricated hydroxyapatite as a support. The hydrophilic group of HA made the scaffold surface more biologically active. Meanwhile, they found that the higher the concentration of hydroxyapatite in the system, the higher the crystallinity, and the lower the degradation rate. Moreover, the degradation products were non-toxic and harmless. Compared with physically mixed hydroxyapatite, hydroxyapatite synthesized in situ has a better proliferation effect on human osteoblast cell line MG-63 [96]. Another example is Guardix-Sol^®®^, a commercially available product made from carboxymethyl cellulose (CMC) and HA, which has been used to prevent tissue adhesion after surgery [97].

#### 3.1.2. Gelatin

Gelatin is a kind of typical natural biomaterial derived from the ECM layer of animals and has outstanding biocompatibility [98,99]. Gelatins can generally be crosslinked with glutaraldehyde, carbon imine, etc. to control their mechanical properties [100]. Gelatin has been applied to design different anti-adhesion barriers. For example, Bober et al. synthesized polypyrrole-gelatin jelly by low-temperature oxidative polymerization in the gelatin system. The experimental results illustrated that the mechanical properties of the reaction mixture were significantly improved with the increase in gelatin content in the reaction mixture. Meanwhile, the cytotoxicity of polypyrrole-gelatin jelly decreased with the increase in gelatin concentration. In the absence of antimicrobial agents, the polypyrrole-gelatin gels also exhibited good antimicrobial activity [101].

#### 3.1.3. Carboxymethyl Cellulose

CMC is synthesized from cellulose and chloroacetic acid through an alkali-catalyzed reaction. CMC is a water-soluble polymer with good biocompatibility and biodegradability; therefore, CMC-based anti-adhesion hydrogel barriers have been well-designed [24,102]. For example, using adihydrazine as the crosslinking agent, Nuraina et al. synthesized a novel graft hydrogel (CMC-g-CMPVA) from carboxymethyl polyvinyl alcohol (CMPVA) and CMC. This smart hydrogel can be used in specific pH environments. The biocompatibility of the hydrogel was observed by tetramethylammonium salt colorimetry and phase-contrast microscopy using a human brain cell as a model. The results showed that the graft with CMC significantly improved the biocompatibility of CMPVA [103].

#### 3.1.4. Chitosan

Chitosan is a natural polysaccharide that has good biocompatibility and biodegradability. In addition, studies have found that chitosan also has excellent free radical-scavenging activity, antibacterial activity, and hemostatic activity [104,105,106]. Compared with other natural polymer materials, chitosan has a longer in vivo decomposition time, making it an excellent anti-adhesion barrier material. Pourjavadi et al. presented a simple and environmentally friendly method for the design of a chitosan/PVA dual network (Chito-PVA-DN) hydrogel with high mechanical properties. The tensile strength and elongation of the Chito-PVA-DN hydrogel increased to 11.8 MPa and 265.6%, respectively. Cell culture experiments showed that the synthesized hydrogels were non-toxic to support cell adhesion and cell growth. Therefore, the addition of natural polymers such as chitosan significantly improved the mechanical properties and biocompatibility of the hydrogels [107].

#### 3.1.5. Alginate

Alginate is abundant in seaweed, with the chemical formula of (C_6_H_8_O_6_)_n_, which is a polysaccharide block copolymer of β-D-manic acid and α-L-gluonic acid. Alginate is non-toxic, and its mechanical properties change with its content; therefore, it has been widely used as a postoperative adhesion barrier [108]. In addition, the anti-adhesion can be improved by loading drugs with anti-adhesion properties [109,110]. In a study, Guan and co-workers prepared a multiple network sodium alginate/krill protein/polyacrylamide hydrogel with a ‘covalent bond ionic complex-hydrogen bond’. The experimental results illustrated that this ionic crosslinked hydrogel was characterized by a unique gradient-distributed three-dimensional porous structure. The mechanical strength of the hydrogel was significantly improved after adding the sodium alginate, and the shape memory effect was observed. Moreover, the crosslinking with sodium alginate was able to facilitate cell adhesion and growth and improve the biosafety of synthetic polymers such as acrylamide [111].

### 3.2. Delivery Performance

Hydrogels have outstanding application prospects in the biomedical field, not only because of their three-dimensional network structure and nanoscale size, but also due to their internal cavity structure. The loading of an anti-adhesion drug into the hydrogel will lead to an enhanced anti-adhesion effect. There are some typical drugs on the market that can prevent adhesions. The first category is anti-inflammatory drugs, including steroids, non-steroidal anti-inflammatory drugs, vitamin E, and low-dose aspirin [112,113]. The second type includes fibrinolytic tissue plasminogen activators, urokinase, and streptokinase [114,115]. The third class is anticoagulants, such as heparin, coumarin, warfarin, direct thrombin inhibitor, and direct thrombin inhibitor [116,117]. What is more, there are other drugs, such as anti-microbial drugs and many others, to be further studied [118].

Typically, hydrogels adapted to drug delivery systems are generally stimulus-responsive hydrogels. They can respond to their environment, and depending on the external environment, the volume and properties of the hydrogel change accordingly. Typical stimuli-responsive hydrogels, such as pH-responsive hydrogels, contain pH-sensitive groups in the polymer network [119,120]. As the pH of damaged tissues changes, such pH-responsive hydrogels may shrink or swell, and drugs encapsulated in the hydrogel can be released when the pH is above or below pKa. Haidari’s team made a pH-sensitive polymethacrylic acid (mAA)-coacrylamide (AAm) hydrogel that can control the delivery of silver nanoparticles to infected wounds. The pH-triggered and controlled release of silver nanoparticles was achieved by changing the pH of the in vivo environment [121].

#### 3.2.1. Anti-inflammatory Drugs

Sirolimus (SRL) is a macrolide antibiotic with immunosuppressive and antiproliferative properties [122]. SRL has anti-angiogenic, anti-proliferative, anti-fibrotic, and anti-inflammatory properties. Maciver et al. investigated the impact of an SRL drug-eluting, hydrogel-impregnated polypropylene mesh in reducing the adhesions to surrounding intestines [123]. In the study, a mouse model of intraperitoneal adhesions showed significant adhesions within one week after surgery, which remained stable over a period of 6 months. When SRL combined with a hydrogel was used to treat postoperative adhesions, the surface area and the severity of adhesions were significantly reduced compared with the control group, demonstrating that the combination of SRL-loaded drug-eluting and hydrogel-impregnated mesh effectively reduced the incidence of postoperative adhesions, as well as the severity and strength of postoperative adhesions [123]. 

Flavonoids are mostly polyphenolic compounds with at least one hydroxyl group attached to a benzene ring that can accept free radicals [124]. Zhang et.al. provided a convenient method for the preparation of supramolecular hybrid hydrogels by the dynamic covalent crosslinking of quercetin (Que), 2-formyl-phenyl boric acid (2-FPBA), and carboxymethyl chitosan (CMCS). The prepared CMCS/2-FPBA/Que (CFQ) composite hydrogel has splendid antioxidant, anti-inflammatory, and antibacterial effects. The good cytocompatibility of the CFQ hydrogel was confirmed by L929 cytotoxicity evaluation, and the ability to resist adhesion of the CFQ hydrogel after cecal defect and abrasion in rats was further evaluated. In comparison with the control group, the tissue adhesion rate of the hydrogel-treated group significantly decreased with the increase in Que concentration. In addition, the sustained release time of C3F0.8Q0.08 hydrogel (3, 0.8, and, 0.08 are the weight percentages of CMCS, 2-FPBA, and Que, respectively, in the composite hydrogel) reached 14 days, which is suitable for clinical trials [125]. 

#### 3.2.2. Tissue Plasminogen Activator

Tissue plasminogen activator (tPA) is a drug that can effectively prevent fibrin clot formation, reduce inflammation, stimulate fibrinolysis, and inhibit fibroblast proliferation. He et al. designed a sustainable delivery system that encapsulated tPA into a thermosensitive hydrogel, forming tPA-hydrogels. The resulting tPA hydrogel is injectable and degradable in vivo after about 4 weeks. The experimental results showed that the concentration of PAI-1 in the peritoneal lavage fluid of rats served with tPA-hydrogel was inferior to that of other groups, resulting in the reduction of fibrin formation. However, there were no remarkable differences in tPA blood concentrations at each time point, proving that the release of anti-adhesion drugs by hydrogel could enhance the effectiveness of preventing adhesion. This thermosensitive hydrogel is a good combination of barrier function and drug therapy for the treatment of severe adhesion [126].

#### 3.2.3. Anticoagulant Drugs

Certain studies have revealed that the use of anticoagulant drugs is a means of preventing postoperative adhesions on account of the thrombus in the abdominal cavity being an irritant that causes adhesion [12,127]. Researchers have found that anticoagulants can inhibit excess thrombus formation by modulating thrombin activity, thereby reducing the conversion of soluble fibrinogen to fibrin [128]. However, there is a potential risk of sustained bleeding when using anticoagulants. Therefore, using such drugs to maintain normal coagulation while effectively limiting postoperative adhesions remains a very challenging issue.

Heparin is the most commonly used anticoagulant drug [129,130]. Low-molecular heparin sodium has been shown to have some effect in preventing postoperative adhesions in a rat model [131]. Soykan et al. performed a dissection on 38 Wistar albino rats to determine the effect of low-molecular weight heparin and hyperbaric oxygen treatment on postoperative adhesions and wound healing. The experimental results illustrated that low molecular heparin sodium reduced abdominal adhesions and had no adverse effect on wound healing. With the combination of hyperbaric oxygen therapy, the potential risk of persistent wound bleeding due to anticoagulant drugs was effectively addressed [127]. Similarly, thrombin inhibitors and platelet inhibitors also showed some prevention of postoperative adhesions [132].

In addition, Zhang et al. designed a kind of HEPA-rin-poloxamer hydrogel (HP hydrogel). They used a cold method to load 17β-estradiol in HP hydrogel (E_2_-HP hydrogel). Therefore, this kind of hydrogel could be administered in the endometrium with slow release. By establishing an intrauterine adhesion (IUA) model, the study showed that the number of glands in the endometrium significantly increased, and the fibrotic area of the endometrium was significantly reduced after treatment with the E_2_-HP hydrogel, suggesting that the hydrogel could significantly promote the regeneration of an IUA-injured endometrium and inhibit apoptosis. In addition, the authors found that E_2_-HP hydrogel was a promising treatment for uterine adhesions by activating the ERK1/2 and MAPKs p38 pathways, and the restoration of IUA was closely related to the upregulation of kisspeptin [133]. 

#### 3.2.4. Anti-microbial Drugs

In abdominal adhesions, it has been shown that some intestinal microorganisms increase the formation of postoperative adhesions. Zindel et al. demonstrated that mesothelial cells are the main source of fibroblasts in adhesions. By RNA sequencing, they found that the activation and transdifferentiation of mesothelial cell ecotopes are driven by EGFR signaling, which is significantly upregulated in the presence of intestinal microbes. During bacterial peritonitis, bacterial contamination promotes adhesion formation via the mesothelial EGFR signaling pathway. In this research, gefitinib, which is a small molecule inhibitor of EGFR, was used to inhibit EGFR phosphorylation and reduce postoperative adhesion formation in vivo [134]. Therefore, antimicrobial agents are likely to be an additional therapeutic target for the prevention of adhesions after laparotomy. 

In addition, pirfenidone (PFD) is an effective anti-fibrotic and anti-microbial agent [135,136]. Ji et al. designed a hyaluronic acid methacryloyl (HAMA) hydrogel loaded with PFD. In vivo studies comparing the PFD-loaded HAMA hydrogel with PFD or HAMA hydrogel only were performed in a rat laminectomy model. The experimental results showed that the PFD-loaded HAMA hydrogel effectively inhibited cell penetration and suppressed the expression of collagen I/III by stabilizing and continuously releasing PFD for the prevention of epidural fibrosis. Thus, this hydrogel can effectively prevent adhesion formation through pharmacological and physical processes [137].

### 3.3. Adhesion Performance

To prevent postoperative adhesion, hydrogels should not only have biocompatibility but also good adhesion properties. The adhesion characteristics of hydrogel to the damaged tissues ensure the stability of the barrier at the site of tissue injury. Standard tissue adhesives are gaining importance in the prevention of postoperative adhesion, mainly because of their distinct advantages, such as their simple operation, lack of disassembly, and effective sealing of air and body fluid leakage. Nevertheless, tissue adhesives also have several disadvantages. For example, fibrin bonding agents and cyanoacrylate have weak tissue adhesion and may cause cytotoxic reactions [138,139]. Researchers have found that the bonding strength between hydrogels and tissues could be enhanced by introducing weak interactions such as dipole–dipole interactions, van der Waals forces, and hydrogen bonds [140,141]. Masaki et al. prepared catechol-hydroxy butyl chitosan hydrogel, which can firmly adhere to the tissue surface by multiple interactions between tissue amino groups and catechol groups [142]. Moreover, the introduction of electrostatic interaction can strengthen the biological adhesion of hydrogels and enlarge their application range. In another study, an electrostatic-mediated antibacterial zwitterionic polyelectrolyte catechol-hydroxybutyl chitosan hydrogel was designed as a barrier to dispute postoperative adhesion. In the hydrogel, amphoteric ions in the hydrogel network (with N^+^(R)_3_ and SO_3_^-^) showed more substantial dipole moments, and the polar groups on the skin can form hydrogen bonds and electrostatic interactions with skin tissue to increase tissue adhesion [25]. 

## 4. New Progress in the Application of Hydrogel in Anti-Postoperative Adhesions

### 4.1. Physical Crosslinking Polymerization

Reversible interactions of molecules can form physical crosslinking hydrogels [143]. Standard physical crosslinking hydrogels can be constituted through interaction between ions, hydrogen bonding, stereoscopic recombination, etc. The main advantage of physical crosslinking is its biomedical safety. Since no chemical crosslinking agents are used during the hydrogel formation, the potential cytotoxicity of unreacted chemical crosslinking agents can be avoided [144,145,146]. In addition, physical crosslinking hydrogels are generally responsive, self-healing, and injectable. Moreover, physical hydrogels are often designed to be biologically active and allow for the encapsulation of live cells and therapeutic molecules.

#### 4.1.1. Ionic–Electrostatic Crosslinked Hydrogel

Ion–electrostatic interactions have been widely used in the construction of hydrogels by physical crosslinking of two molecules with opposite charges [147,148,149,150]. For instance, naturally extracted polysaccharides such as alginate containing glucuronic acid residues and mannuronic acid can be crosslinked with divalent cations such as magnesium (Mg^2+^), barium (Ba^2+^), and calcium (Ca^2+^) [151]. The glucuronic acid residue of one polymer forms a physical crosslink with another glucuronic acid residue of the adjacent polymer chain and forms a hydrogel. Moreover, certain macromolecules could also interact with each other to form a polyelectrolyte complex. For example, chitosan can form polyelectrolyte complexes through electrostatic interactions between its cationic amino groups and various naturally occurring anionic polyelectrolytes (such as DNA, pectin, and gelatin) [152,153,154]. In addition, chitosan may have a mutual effect with several synthetic polymers such as polyphosphoric acid, polyacrylic acid, and polylactic acid. The hydrogels generated by these polyelectrolyte complexes can be regulated by many factors, including the soluble microenvironment of the polymers, the mixing ratio and amount of each polymer, and the charge density of the polymers. The superiority of this type of hydrogel is its good self-healing ability. Therefore, a broken physical network of the hydrogel due to high stress can be re-formed when the stress is relieved. Nevertheless, the mechanical strength of ion–electrostatic crosslinked hydrogels is limited [155,156,157]. 

**Figure 2:**
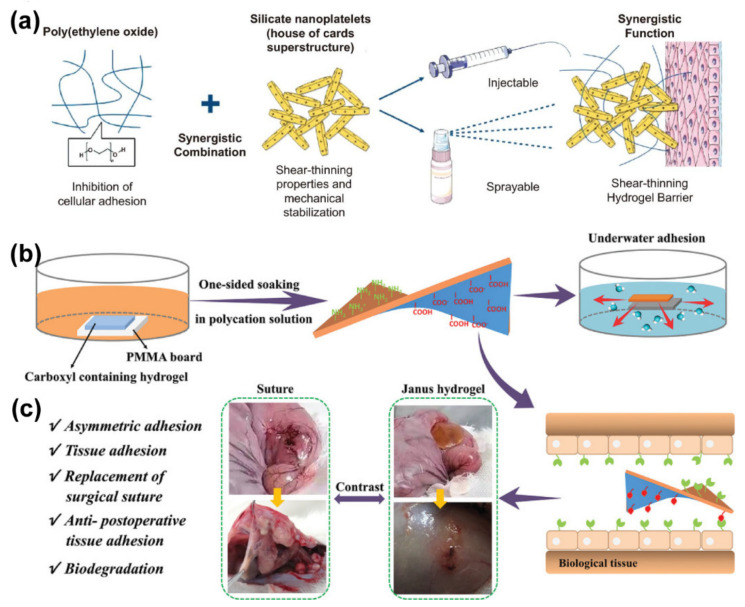
Some samples of ionic–electrostatic crosslinked hydrogels. (**a**) Schematic representation of the sprayable shear-thinning hydrogel barrier formulation and delivery methods. Reprinted with permission from Ref. [158]. Copyright 2021 The Author(s). (**b**) Schematic representation of Janus hydrogel formation by gradient polyelectrolyte complexation with application in underwater adhesions and (**c**) Janus hydrogel application in the treatment of gastric perforation in rabbits. Reprinted with permission from Ref. [159]. Copyright 2020 Wiley-VCH GmbH.

In a study, Khademhosseini et al. developed a shear-thinning hydrogel consisting of polyethylene oxide and silicate nanosheets that can be delivered by injection and spray methods (Figure 2a). The attraction between the positively charged edge and the negatively charged surface of the silicate nanosheets leads to the spontaneous formation of the superstructure, which allows for self-assembly in water and provides an organized tortuous network to prevent the transport of molecules and colloidal particles. Therefore, the silicate nanosheets were combined with polyethylene oxide to prepare hydrogels with good minimal cell adhesion, low immunogenicity, and anti-fouling properties or protein adsorption for preventing postoperative adhesions [158]. In another study, a unilateral impregnation method is proposed by Liu et al. for the preparation of novel Janus hydrogel wet gels by the gradient electrostatic complexation of negatively charged carboxylated hydrogels with cationic oligosaccharides (Figure 2b). The Janus hydrogel is adhesive on one side and non-adhesive on the other side, which makes it a good candidate to prevent postoperative adhesion. In the process of synthesis, the hydrogel was separated by the electrostatic complex method, which increased the hydrophobicity and drainage capacity. Thus, it showed mild complexation on the surface even in water, while instantly producing strong adhesion to various wet tissues. This hydrogel is therefore particularly suitable for visceral surfaces where adhesion is likely to occur after surgery. Animal experiments fully demonstrated that one side of the Janus hydrogel has remarkable biocompatibility and biodegradability, which effectively prevents postoperative adhesions (Figure 2c). Hydrogels formed by the complexation of gradient polyelectrolytes create a new direction for tissue and organ repair and the prevention of postoperative adhesion [159]. 

#### 4.1.2. Hydrogen Bonds Crosslinked Hydrogel

**Figure 3:**
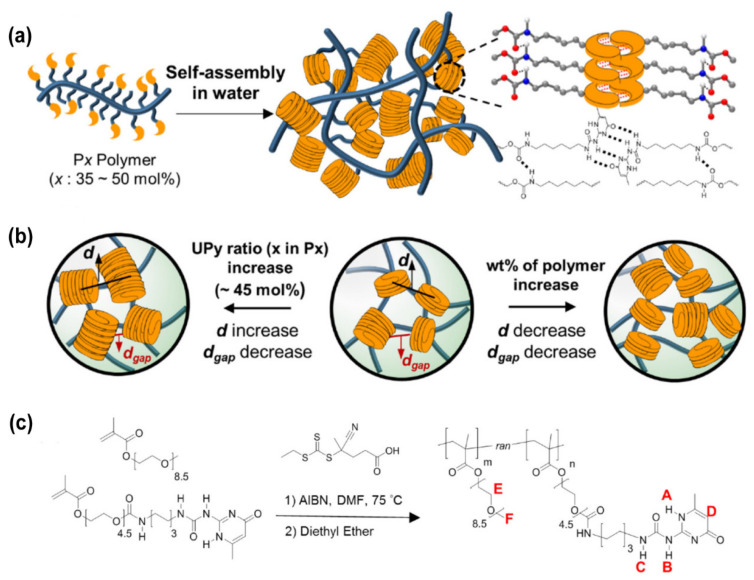
(**a**) A general view of the self-assembly of P(PEGMA-co-(PEGMA-UPy)) polymers in the aqueous solution. The yellow discs indicate the UPy-UPy dimer. (**b**) Schematic representation of the interdomain distance (*d*) and the characteristic gap distance (*d_gap_*) as a function of the UPy fractions and the polymer weight fraction in the hydrogel. (**c**) Schematic representation for the synthesis of P(PEGMA-co-PEGMA-UPy)) polymers. Reprinted with permission from Ref. [164]. Copyright 2021 American Chemical Society.

Hydrogen bonds can stabilize secondary structures during the formation of the polypeptide or agarose-based hydrogels [160]. During the hydrogel formation, hydrogen bonds can be formed between pyrrole, urea, hydroxyl groups, amides carbazoles, and carboxylic acids, and they can also have a mutual effect with electron-donor groups such as pyridine and imidazole groups [161,162,163]. However, hydrogen bonds alone are usually not sufficient to support hydrogel formation. For example, powerful networks can be shaped using ureidopyrimidinone (UPy), which creates multiple polyvalent hydrogen bonds. Based on the dimerization reaction of quadruple hydrogen bonds among the random amphiphilic copolymers containing about 35–50 mol. % of UPy, Choi et al. designed a kind of antifreeze and self-healing hydrogel (Figure 3). Owing to the π−π interactions, the UPy-UPy dimers can stack with each other, resulting in the physical crosslinking hydrogels when the pH > 8. In addition, these π−π interactions substantially enhanced the toughness of the hydrogel and made the hydrogel more stable [164].

**Figure 4:**
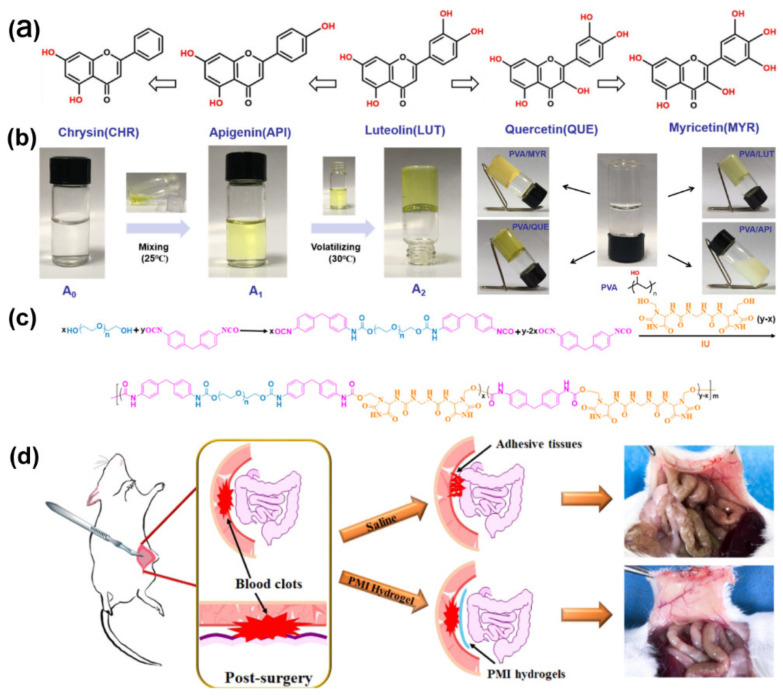
(**a**) Structure of chrysin (CHR), apigenin (API), luteolin (LUT), quercetin (QUE), and myricetin (MYR). (**b**) Schematic diagram of synthetic PVA/QUE hydrogel. A_0_, PVA solution; A_1_, PVA/drug water-ethanol mixture; A_2_, PVA/drug hydrogel and the schematic diagram of the synthesis of PVA/API, PVA/LUT, PVA/QUE, and PVA/MYR hydrogel. Reprinted with permission from Ref. [165]. Copyright 2021 American Chemical Society. (**c**) Synthetic procedures of the PMI polymers; (**d**) general scheme of PMI-1 hydrogel as a physical barrier against adhesion. Reprinted with permission from Ref. [166]. Copyright 2020 American Chemical Society.

The preparation of hydrogel carriers for hydrophobic drugs is still a considerable challenge. Fortunately, hydrogels made by hydrogen bonding can be loaded with drugs. For example, Hu et al. designed a hydrophobic drug-loaded hydrogel, working mainly through hydrogen bonding. The hydrophilic PVA and some hydrophobic compounds (lignocaine and quercetin) actuate the formation of hydrogen bonds by the evaporation of ethanol to produce supramolecular hydrogels (Figure 4a,b). The results suggested that the formation of the hydrogel is a supramolecular self-assembly process. Similarly, due to the hydrogen bonds formed between the drug and PVA being non-covalent and reversible, this hydrogel has appealing self-healing properties. The author established an adhesion model of lateral wall-caecum wear in rats, and the experimental results showed that this hydrogel exhibited excellent efficiency and safety in preventing postoperative adhesions [165]. 

In another study, Wang et al. designed a kind of supramolecular hydrogel called a PMI hydrogel that was constituted with imidazolidinyl urea (hydrogen bonding-reinforced factor), methylene diphenyl 4, 4-diisocyanate, and poly(ethylene glycol) with high toughness and biodegradability, to serve as a physical barrier for abdominal adhesion prevention. The hydrogel was formed by the multiple hydrogen bonds among urethane groups and imidazolidinyl urea and the hydrophobic interaction of the benzyl ring of methylene diphenyl 4,4-diisocyanate (Figure 4c). This research mainly investigated the effect of imidazolidinyl urea content and the molecular weight of poly(ethylene glycol) on the shape of memory behavior, energy dissipation efficiency, and mechanical properties. By constructing a mouse abdominal wall defect model, the excellent antiadhesion effect of the hydrogel was confirmed (Figure 4d) [166].

### 4.2. Chemical Crosslinking Polymerization

#### 4.2.1. Chemical Crosslinking Hydrogel

**Figure 5:**
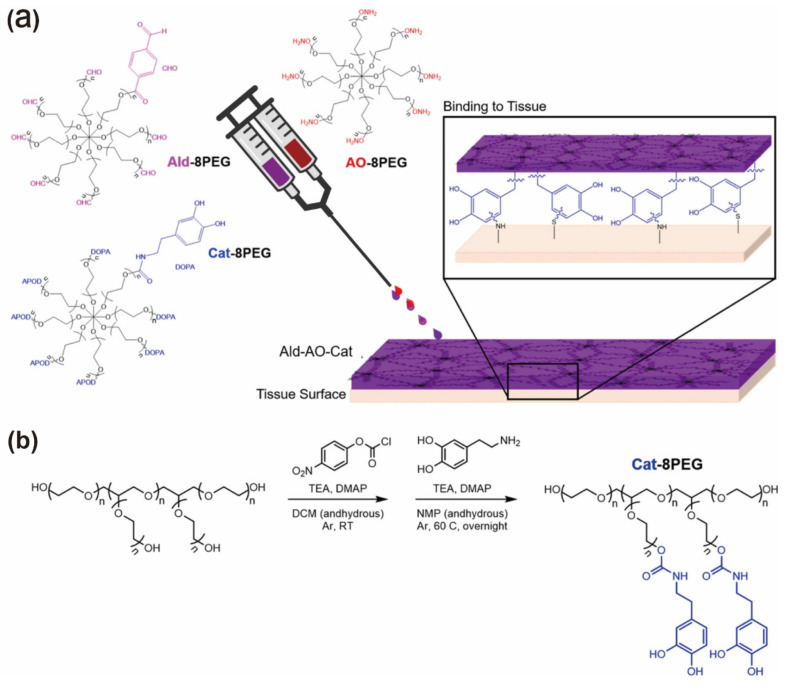
(**a**) Schematic diagram of oxime hydrogel formation using three distinct 8-arm star PEG polymers (AO-8PEG, Ald-8PEG, Cat-8PEG). (**b**) Schematic diagram using a two-step method to synthesize the Cat-8PEG. Reprinted with permission from Ref. [142]. Copyright 2021 The Author(s).

In chemical crosslinking hydrogels, polymer chains usually form covalent bonds with each other. In comparison with physical crosslinking hydrogels, chemical crosslinking hydrogels generally exhibit higher stability, remarkable mechanical property, and adjustable degradation behavior under physiological conditions [47]. Up to now, various kinds of crosslinking methods, including photoinitiated hydrogels, free radical polymerization-induced crosslinking, enzymatic reaction, and ‘Click’ reactions have been reported [64,68,71]. Masaki et al. proposed a hydrogel barrier consisting of oxime-crosslinked polyethylene glycol and catechol inclusion to improve cardiac retention and prevent pericardial adhesion. The ternary system is formed by mixing aldehydes, aminooxy, and Cat-functionalized PEG to form a hydrogel (Ald-Ao-CAT) (Figure 5). Compared to the original Ald-Ao gel formulation that consists of aminooxy-terminated PEG and aldehyde-terminated PEG, Ald-Ao-CAT has wonderful tissue retention, minimal swelling, degradation kinetics, and superior mechanical properties. The authors showed in live rat and pig models that the hydrogel was cytocompatible, resistant to cell adhesion, and led to a reduction in adhesion severity [142].

#### 4.2.2. Photoinitiated Hydrogel

Photoactivated crosslinking has been widely used in therapeutic or cytokine-embedding hydrogel formation [167,168,169]. The advantages of photoactivated crosslinking include the fact that the hydrogel can be formed quickly under mild conditions, and the mechanical properties of the hydrogel are tunable by controlling the crosslinking conditions [170]. Moreover, the exact crosslinking point can be selected, since the photo-polymerization takes place under light, and only the irradiated region is involved in the crosslinking reaction. Photoinitiated polymerization is associated with the presence of unsaturated groups and cell-compatible photoinitiators that can absorb specific light at different wavelengths, including red light (750–810 nm), visible blue-violet light (405–550 nm), and ultraviolet light (250–370 nm) [171,172].

**Figure 6:**
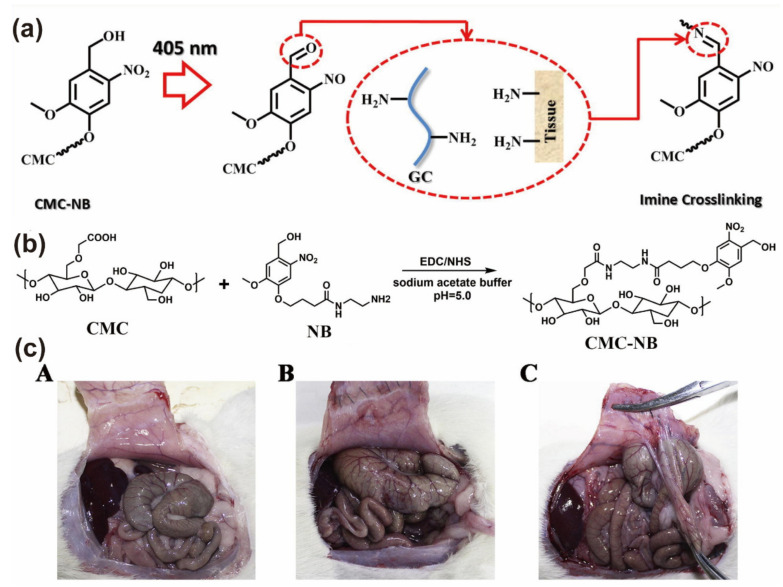
(**a**) Schematic illustration of CNG hydrogel for postoperative anti-adhesion in 405 nm. (**b**) The diagram of synthetic route and structure of CMC-NB hydrogel. (**c**) Anti-adhesion efficacy assessment by the abdominal wall-cecum defect model of SD rats. Gross observation of the abdominal wall-cecum sites in SD rats treated by CNG1 hydrogel (A), HBC hydrogel (B), or saline rinsing (C) on the 14th day after surgery. Reprinted with permission from Ref. [24]. Copyright 2017 Acta Materialia Inc.

On account of the photo-induced imine crosslinking reaction, Wang et al. developed a CNG hydrogel that is composed of glycol chitosan and o-nitrophenyl alcohol (NB)-modified CMC (CMC-NB) as an anti-adhesive barrier material (Figure 6a). Under UV irradiation, CMC-NB generates aldehyde groups that react with amino groups on the tissue surface to form a hydrogel barrier and covalently attach to the tissue surface (Figure 6b). A series of experiments showed that CNG hydrogels have outstanding tissue adhesion strength, cytocompatibility, and an excellent anti-adhesive effect. Moreover, the bond strength of CNG hydrogels increased with the increase in CMC-NB content. The authors established a rat lateral wall defect-appendage abrasion model and experimentally showed that the CNG hydrogel-treated group ((A) in Figure 6c) showed significantly lower tissue adhesion compared with the commercially available hydrogel group ((B) in Figure 6c) and control group ((C) in Figure 6c). In addition, the CNG hydrogel degraded within 14 days and had no side effects on wound healing [24].

**Figure 7:**
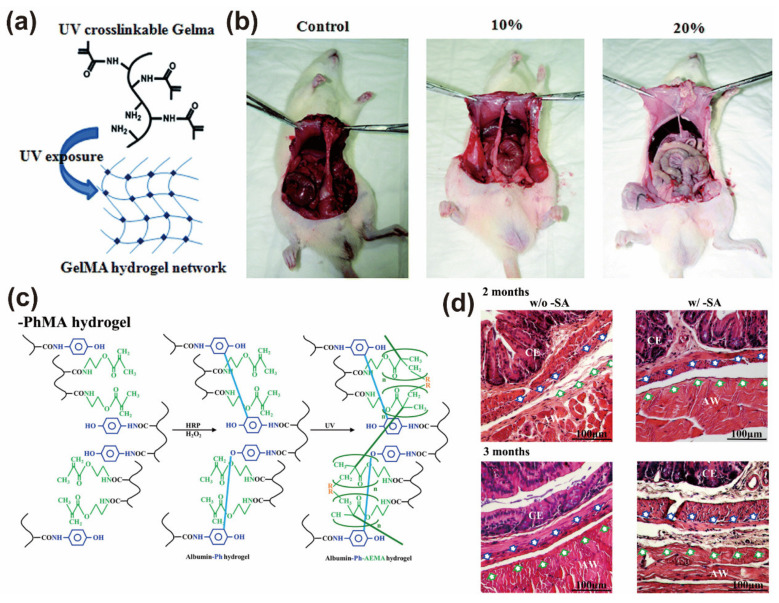
(**a**) Schematic illustration of GelMA hydrogel network. (**b**) The evaluation of a rat cecum abrasion model after 14 days. The results showed that 20% GelMA hydrogel film had the best anti-adhesion property. Reprinted with permission from Ref. [173]. Copyright 2011 RSC publication. (**c**) Schematic illustration of the synthesis of BSAPhMA albumin-based hydrogels. (**d**) H and E-stained sections of the abdominal and cecum sidewalls of mice treated with BSAPhMA hydrogels without salicylic acid and with salicylic acid at 2 and 3 months after treatment. The blue arrows and green arrows point to the repaired mesothelial layer of the abdominal cecum and wall, respectively. Reprinted with permission from Ref. [174]. Copyright 2021 American Chemical Society.

In another study, Cui and co-workers synthesized a photo-crosslinked gelatin-methacrylate (GelMA) hydrogel to prevent the formation of intragastric adhesions. Methacrylate (MA) was added into a gelatin solution, and it reacted to form a photo-crosslinked gelatin precursor. A water-soluble initiator 2-hydroxy-40-(2hydroxyethoxy)-2-methylpropiophenone was added as a photoinitiator that can be photo-crosslinked with GelMA under UV light to form UV polymerized hydrogel (Figure 7a). The obtained GelMA-based hydrogel film has the advantages of simple operation, non-toxic degradation, and a longer-term good barrier effect (up to one month). After intraperitoneal implantation of GelMA hydrogel, the tissue adhesion formation in experimental rats was meaningfully reduced compared with the control group (Figure 7b) [173]. Chen et al. designed a kind of hydrogel that is sensitive to H_2_O_2_ and can coverage to the wound surface and provide good adhesion. At the same time, a photosensitive crosslinking agent (2-hydroxy-1-(4-(hydroxyethoxy) phenyl)-2-methyl-1-propanone) was added to exploit a dual in situ crosslinked albumin-based hydrogel. The authors concluded that if the crosslink density and stiffness of the hydrogel can be further adjusted after the completion of gelation, it can make the application of this hydrogel more flexible. Therefore, the authors functionalized the albumin molecules with phenolic and methacrylate groups and crosslinked them in multiple steps to finally form BSAPhMA hydrogels (Figure 7c). This hydrogel can be spatially post-adjusted via transdermal exposure to light to release the salicylic acid drugs. The experimental results illustrated that the albumin hydrogel successfully prevented postoperative tissue adhesion and reduced the formation of fibrous tissue due to the good biocompatibility and persistence of the hydrogel (Figure 7d). Therefore, the serum albumin hydrogel with a multi-stage drug release profile can serve not only as a physical barrier but also as a multi-stage drug carrier to reduce local immune response and promote tissue regeneration [174].

#### 4.2.3. ‘Click’ Chemically Reactive Hydrogel

**Figure 8:**
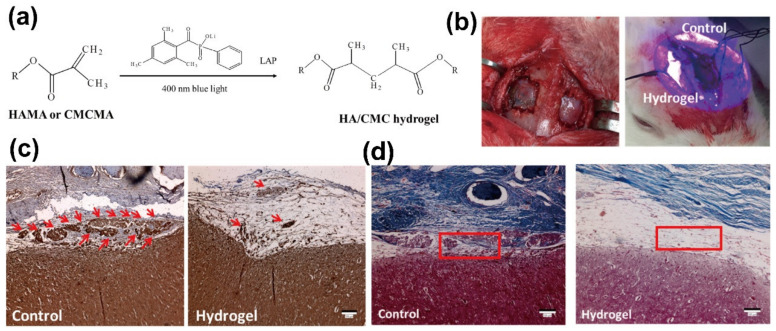
(**a**) Diagram of HA/CMC hydrogel synthesis using the photocrosslinking reactions. (**b**) The bilateral defects were established on the rabbit’s skull; one defect was treated with hydrogel, and the other was not treated. (**c**) The immunohistochemical staining of vimentin (red arrows mean fibroblasts synthesized vimentin) and (**d**) the H and E staining image in adhesion tissue of the control group and hydrogel group, bar = 100 μm. Reprinted with permission from Ref. [169]. Copyright 2022 The Author(s).

Currently, ‘click chemistry’ is being frequently used for the preparation of functional hydrogels. During hydrogel production, click chemistry has the following characteristics: high yield, less harmless byproducts, strong reaction stereoselectivity, simple reaction conditions, and so on [175]. Many functional groups can be involved in click chemistry to form complex polymer hydrogels. This section briefly introduces the Schiff base method. Under physiological conditions, Schiff base formation usually occurs between amino and acetaldehyde to produce imine bonds [176]. Because there is a dynamic equilibrium between the aldehyde and amine reactants, these bonds can also be regarded as pseudo-covalent bonds. Due to the continuous uncoupling and re-coupling of the imine bonds in the hydrogel network, the hydrogel has an obvious self-repair ability and can be used to construct injectable and self-healing hydrogels. Moreover, aldehyde groups can easily and firmly adhere to tissues or organs [177,178,179]. Li et al. proposed a bionic hybrid hydrogel composed of oxidized HA, glycolic chitosan, and conditioned medium (CM) derived from stem cells membrane. The hybrid hydrogel was formed mainly by the reversible Schiff base reaction and therefore possessed good self-healing ability and injection properties (Figure 8a). In addition, due to its high density of positive charge and characteristic viscosity, the hydrogel showed excellent hemostatic, anti-infection, and tissue adhesion capabilities. Moreover, the biological factors contained in CM can be released from the hydrogel in a controlled manner, providing significant potential for hepatocyte proliferation and tissue regeneration. The authors used a rabbit dural defect model for in vivo studies of this hydrogel (Figure 8b). The experimental results illustrated that the hydrogel-treated group showed an effective reduction in intra-abdominal adhesions and a significant improvement in hepatocyte proliferation and tissue regeneration (Figure 8c,d) [169].

## 5. Conclusions

Hydrogels have admirable biocompatibility and biodegradability. They can load different drugs and control the drug release kinetics. In addition, hydrogels have an excellent antifouling ability; therefore, hydrogels can prevent the adhesion of proteins and cells on the surface. In this review, the advantages, applications, and recent advances of hydrogels as postoperative adhesives are reviewed. Some typical hydrogel classifications and synthesis methods were also mentioned. The biocompatibility, biodegradability, and delivery performance have facilitated the application of hydrogels to prevent tissue adhesion. However, the degradation rate of certain kinds of natural polymers is fast, resulting in insufficient anti-adhesion. In contrast, the degradation velocity of synthetic polymers is slower than natural polymers and thus has a longer retention duration, which can facilitate the complete prevention of postoperative adhesions. The state-of-the-art progress in the application of hydrogels in anti-postoperative adhesions was finally summarized. Although various research results have been reported, designing a smart stimuli-responsive hydrogel remains a major challenge in the design of anti-adhesive hydrogels. It is hoped that in the future, hydrogels with outstanding biocompatibility and mechanical properties will be used as anti-adhesive barriers.

## Data Availability

Not applicable.
